# Patient perspectives on cervical cancer screening interventions among underscreened women

**DOI:** 10.1371/journal.pone.0277791

**Published:** 2022-12-01

**Authors:** Andrea C. Des Marais, Noel T. Brewer, Suzanne Knight, Jennifer S. Smith

**Affiliations:** 1 Department of Epidemiology, Gillings School of Global Public Health, University of North Carolina at Chapel Hill, Chapel Hill, North Carolina, United States of America; 2 Department of Health Behavior, Gillings School of Global Public Health, University of North Carolina at Chapel Hill, Chapel Hill, North Carolina, United States of America; 3 Lineberger Comprehensive Cancer Center, University of North Carolina at Chapel Hill, Chapel Hill, North Carolina, United States of America; 4 Cabarrus Health Alliance, Kannapolis, North Carolina, United States of America; Johns Hopkins Bloomberg School of Public Health, UNITED STATES

## Abstract

**Background:**

Cervical cancer is highly preventable with regular screening, yet over 4,000 women die from it annually in the United States. Over half of new cervical cancer cases in the U.S. are attributable to insufficient screening.

**Methods:**

Participants were 23 low-income, uninsured or Medicaid-insured women in North Carolina who were overdue for cervical cancer screening according to national guidelines. Semi-structured interviews examined perspectives on barriers to cervical cancer screening and on interventions to reduce these barriers. We also elicited feedback on three proposed evidence-based interventions: one-on-one education, coupons to reduce out-of-pocket costs, and self-collection of samples for detection of high-risk human papillomavirus (HPV) infection, the primary cause of cervical cancer.

**Results:**

Reported barriers included high cost, inconvenient clinic hours, lack of provider recommendation, poor transportation, difficulty finding a provider, fear of pain, and low perceived need. Participants suggested interventions including reducing cost, improving convenience through community-based screening or extended clinic hours, strengthening provider recommendations, and providing one-on-one counseling and education outreach. HPV self-collection was most frequently selected as the “most helpful” of 3 proposed interventions (n = 11), followed by reducing out-of-pocket costs (n = 7) and one-on-one education (n = 5).

**Conclusion:**

Cost was the most reported barrier to cervical cancer screening, although women experience multiple simultaneous barriers. Novel interventions such as HPV self-collection promise to reduce some, but not all, barriers to primary screening. Interventions that work on reducing multiple barriers, including obstacles to receiving follow-up care, may be most effective to prevent cervical cancer among these high-risk women.

## Introduction

Wide scale implementation of primary screening for early detection and treatment of cervical precancerous lesions has dramatically decreased the burden of cervical cancer in the United States (U.S.) over the last half-century [1]. Even so, an estimated 14,100 U.S. women will develop this preventable cancer in 2022 and 4,280 will die from it [1]. Women who are Black, Latina [1], uninsured [2], and low-income [3] are most likely to be diagnosed with cervical cancer, and mortality rates are nearly double among Black women compared to White women [4].

Over half of new cases of cervical cancer in the U.S. can be attributed to insufficient screening [5]. Current screening guidelines recommend Pap testing alone every three years for individuals aged 21 to 29, and Pap testing alone every three years or in combination with HPV testing every five years for individuals aged 30 to 65 years [6]. One-fifth of U.S. women report being overdue for screening, and actual rates of being overdue are most likely higher, as self-report has been shown to over-estimate cancer screening [7, 8]. A wide range of factors are associated with a lower likelihood of completing cervical cancer screening, including lack of health insurance, low income, residing in rural or economically deprived areas, lack of provider recommendation, having a male provider, low health literacy, and low knowledge about cervical cancer [9–11]. Additional reported barriers to screening include embarrassment, fear of cancer, mistrust of the medical system, poor rapport with the provider, physical discomfort from the procedure, lack of time, transportation, and inconvenient clinic hours [12–14].

A range of interventions have been evaluated to increase cervical cancer screening. An evidence review by the U.S. Community Preventive Services Task Force (CPSTF) recommends three individual-level (“client-oriented”) evidence-based interventions to increase cervical cancer screening: *small media* that provide information to educate and motivate women, *one-on-one education* delivered by healthcare workers or trained lay people in medical or community settings, and *client reminders* informing women that they are due or overdue for screening [11, 35]. The CPSTF found sufficient evidence to recommend *evaluating provider performance* in offering and delivering cervical cancer screening to increase provider delivery [15]. *Reducing out-of-pocket costs* and *reducing structural barriers* are recommended for breast and colorectal cancer screening, though the CPSTF review found too few studies to make a recommendation for cervical cancer [15].

Another innovative approach to increase cervical cancer screening uptake is self-collection of samples to test for high-risk human papillomavirus (HPV), the primary cause of cervical cancer [16, 17]. Offering HPV self-collection to women overdue for in-clinic screening has been found to increase cervical cancer screening uptake compared to referral to in-clinic screening [18] and can address many logistical and psychological barriers to screening [14]. In two studies with under-screened, low-income women in North Carolina [19–21], our team has found that mail-based HPV self-collection in this population is feasible, is well-accepted, and performs well for high grade cervical intraepithelial neoplasia or cancer (CIN2+) detection.

Here, we present infrequently screened women’s views on barriers to cervical cancer screening and interventions designed to increase screening. In addition, these women provided their own ideas for interventions to increase cervical cancer screening, as well as their reactions to three evidence-based interventions.

## Materials and methods

### Participants and recruitment

To enroll in the study, women had to be 25 to 65 years of age, biologically female, infrequently screened (unscreened by Pap test in four years or more), without hysterectomy, and uninsured or publicly insured. We recruited participants from December 2011 to March 2012 in seven North Carolina counties selected based on a relatively high incidence of cervical cancer and low economic development [22, 23]. Women were recruited through flyers, by word of mouth, and in-person at Departments of Social Services and Health Departments.

### Procedures

We developed a semi-structured interview guide, which was pilot tested with two women at a local free clinic and revised for clarity. Interviews explored participants’ knowledge of and experiences with cervical cancer screening, barriers to screening, and healthcare access. To identify barriers to screening, we first asked the open-ended question, “What are some things that have gotten in the way for you [getting a Pap smear]?” then asked participants to identify from this list the “biggest thing that keeps you from getting a Pap smear.” We asked participants to propose interventions to address their barriers to screening, and to provide their reactions to three evidence-based interventions (Table 1). The description for the “helper” was based on lay terms to introduce a one-on-one education intervention, for example, someone who might “talk to you about some of the things that make you nervous” to get a Pap test. We developed the “coupon” to describe a cost-reduction intervention, with “a phone number to call to find out where to you can get a low-cost or free [Pap smear].” The third intervention, “self-test,” described self-collection of samples for HPV testing, using a mailed kit, with the sample mailed back to our study team and results delivered by phone. After describing each intervention, we asked the participant to tell us her “gut reaction,” to identify specific things she liked and disliked about the intervention idea, and to state whether she thought the intervention would help or motivate her to get screened. After we discussed the three interventions, we asked each woman to select the ones she felt would be “most helpful” and “least helpful”.

**Table 1:** Simple descriptions of evidence-based interventions.

**“Helper” (One-on-One Education)** The first idea is to have someone help you with whatever you need to do to get a Pap smear. This “helper” person can help you understand what makes it hard to get a Pap smear and then help make it easier. They might tell you about a place where you can get a free or affordable Pap smear, help you make an appointment to get a Pap smear, or talk to you about some of the things that make you nervous about getting one.
**“Coupon” (Reducing Out-of-Pocket Costs)** The next idea is to send you a coupon in one of those envelopes full of coupons we all get in the mail. The coupon says why to get a Pap smear and has a phone number to call to find out where to you can get a low-cost or free one.^a^
**“Self-Test” (Self-Collection for HPV Testing)** The third idea is a kind of Pap smear you can do at home. The test could be given to you in person or mailed to you at home. To take it, you put a brush or piece of plastic inside you, like a tampon, turn it around a few times, and take it out. Then you mail your sample to a lab and they call you with your results. If the self-test says you have a problem, then you need to go get a Pap smear.

^a.^ Other locations for the coupon distribution were suggested in probes, e.g., “What if the coupon came in the newspaper?”

Two researchers (AD and SK) with graduate level training in qualitative methods conducted 23 semi-structured interviews: 16 in person and seven by phone. Interviews ranged from 45 to 75 minutes in length. Twenty were conducted in English and three were conducted in Spanish by a bilingual interviewer. We selected a target sample of 20 interviews *a priori*, based the fact that our research questions were fairly specific and all participants were within the single category of underscreened women [24]. We interviewed an additional three Spanish-speaking women to improve the diversity of our sample. All interviews were recorded, with notes taken during the interviews. Participants received a $30 gift card, education on cervical cancer prevention, and information for clinics in their area offering free and reduced-cost cervical cancer screening.

Procedures were approved by the Institutional Review Board of the University of North Carolina at Chapel Hill.

### Analysis

Interviews were recorded, then detailed notes were taken from recordings and independently coded by two researchers (AD and SK) using principles of applied thematic analysis [25]. First, *a priori* topical codes were developed based on the interview guide and applied to interview notes. After independently coding 2 interviews, the researchers met to discuss application of the topical codes, resolve discrepancies, and adjust the codebook as needed. The two coders then applied topical codes to all interviews. Upon reviewing the data relevant to each topic, the research team developed content codes. Researchers (AD and SK) double-coded one fourth of the interviews, and met to discuss and assess agreement before coding the remaining interviews. Spanish interviews were coded by a bilingual researcher (AD) without translation. Finally, data were compiled into a matrix to identify themes within each topic and generate counts [26]. All authors met regularly during the analysis process to discuss codes, categories, and emergent themes.

For barriers to screening, we identified 11 emergent categories, then assigned these categories to three themes based on an ecological understanding of determinants of health behavior: economic/structural (organizational/community/policy), provider (interpersonal), and individual [27]. We define *economic/structural barriers* as those emerging from the cost of care and from challenges of navigating health care and transportation systems, *provider barriers* as those emerging from a health care provider’s behavior, and *individual barriers* as those emerging from the participants’ beliefs and attitudes. We noted how many times each barrier was mentioned and was identified as a primary barrier to screening. Suggested interventions and feedback on the three proposed interventions were similarly coded to identify emergent common themes. We assigned interventions proposed by the participants into categories used by the CPSTF reviews: increasing community access (economic/structural level), increasing provider delivery (provider level), and increasing community demand (individual level) [28].

## Results

### Participants

Nine of the 23 participants were Black (39%), seven White (30%), four American Indian (17%), and three Latina (13%) (Table 2). The Latina participants were all first-generation immigrants, and two were monolingual Spanish-speakers. Two thirds (65%) of our sample were uninsured and one third (35%) had Medicaid. One fourth (26%) had not completed high school and about half (52%) had completed some college. All participants were overdue for screening by current recommendations (4 or more years since prior Pap alone), with time since last Pap ranging from 4 to 20 years (median 5 years). Age of participants ranged from 27 to 65 (median 42). Two participants reported having known women who died of cervical cancer: a participant’s mother and a friend’s wife.

**Table 2:** Participant characteristics.

	n (%)
**Race/Ethnicity**	
African American/Black	9 (39)
American Indian	4 (17)
Latina	3 (13)
White	7 (30)
**Insurance**	
Medicaid	8 (35)
Uninsured	15 (65)
**Education**	
Some high school or less	6 (26)
Completed high school	3 (13)
Some college	12 (52)
4 year degree	2 (9)
**Years since last screening** (median, range)	5 (4–20)
**Age** (median, range)	42 (27–65)

### Barriers

In response to the open-ended question, “What are some things that have gotten in the way for you [getting a Pap smear?],” all but one woman reported multiple barriers (Table 3).

**Table 3:** Barriers reported by participants in open-ended response.

	Primary barrier n (%)^a^	Named barrier^b^ n (%)
**Economic/ Structural Barriers**		
Cost / difficulty finding affordable provider	15 (65)	16 (70)
Inconvenient clinic hours and/or long waits	-	5 (22)
Poor transportation	1 (4)	3 (13)
Trouble finding provider accepting new Medicaid patients	1 (4)	1 (4)
**Provider Barriers**		
Lack of provider recommendation or offer	1 (4)	4 (17)
Fear of pain or embarrassment due to previous exam experience	1 (4)	4 (17)
**Individual Barriers**		
Discomfort with unknown provider	4 (17)	6 (26)
Low priority	2 (9)	6 (26)
Low perceived need / lack of understanding of purpose	1 (4)	5 (22)
Does not seek preventive care in general	1 (4)	8 (34)
Embarrassment receiving from current provider (male; “know them too well”)	1 (4)	2 (9)

^a.^ Percentages sum to greater than 100% because of multiple responses from participants

^b.^Only barriers named as primary barrier and/or named as a secondary barrier by 3 or more women are included. Not listed: Fear of results (2), lack of English (2), general mistrust in health screenings (1), and embarrassment due to weight (1).

### Economic/Structural barriers

Among the 16 uninsured women, all mentioned cost as a barrier, and cost was the primary barrier for all but one uninsured woman. Cost was not mentioned by public insurance recipients. In the cost category, we included uncertainty regarding cost, difficulty getting quotes from providers for the cost of an appointment, rejection from a charity program due to inability to prove income, and clinic requirements for payment in-full at the time of service. Several structural barriers were also related to economic constraints: reliance on inadequate public transportation, difficulty finding a provider accepting new Medicaid patients, and the tendency for free clinics to have limited hours and long wait times. Three women described experiences of previously being “shot down” when trying to get a free or low-cost Pap test, then giving up. The two monolingual Spanish-speaking participants did not want to call to schedule an appointment due to language barriers.

### Provider barriers

Five uninsured women and six publicly insured reported seeing a healthcare provider annually or more frequently for treatment of a chronic condition, such as diabetes. Four of these participants reported that their provider had not recommended that they complete a Pap test, or that their providers asked if they had been screened but did not offer or tell them how to get screened. Some women described an acutely painful (n = 3) or emotionally uncomfortable (n = 1) previous experience with cervical cancer screening. We classified these experiences as provider barriers because all of these participants reported that their subsequent aversion to screening could have been reduced had the provider explained the procedure more clearly in advance or talked with the patient about her discomfort or subsequent reluctance to screen. For example, one woman otherwise liked her current provider but received a very painful Pap from her. This participant stated that she would be willing to get another Pap if her doctor had discussed the experience with her at the time or had since asked her why she was reluctant to get another one. One woman was embarrassed to get a pelvic exam again because a nurse had commented at a previous appointment that the participant “might smell”.

### Individual barriers

Though only eight women understood that a Pap test screened for cancer, all but two were aware that they “should” get screened. Six women expressed hesitancy to see a new provider based on two factors: fear of seeing a male provider, and concern that an unknown provider might be “rough.” Three women reported that they did not want to receive a pelvic exam from their *current* provider, two because he was male, and one because she knew the clinic nurses socially.

Six women acknowledged that cervical cancer screening was not a high priority. They mentioned competing demands for their time and money, as well as a general tendency to put off the examination, as one participant said, “it’s on the *gonna do* list.” Six uninsured women and two with Medicaid mentioned not seeking preventive care in general, instead going to the doctor only when “something is wrong.” Two reported that they just “hate going to the doctor.” Three women reported that they did not know whether their mothers had received regular screenings and therefore never “got in the habit” of doing it themselves. One woman reported fear of a “bad result,” one avoided health screenings due to perceived risk of undergoing unnecessary treatment, and one felt embarrassment due to her weight.

Five women acknowledged that they would probably try harder to overcome other barriers to screening if the procedure itself were not so uncomfortable. As one participant stated, she “would have found a way to work it out if [she] really wanted to”.

### Interventions suggested by participants

After discussing barriers to screening, we asked each participant to come up with her own ideas for what would help to overcome her barriers (Table 4). The suggested interventions largely corresponded to women’s reported barriers.

**Table 4:** Interventions proposed by participants.

**Increase Community Access (Structural Level)–Suggested by 14 women**
Reduce out-of-pocket cost of exam
Allow for payment plans
Improve insurance coverage in general
Improve transportation
Hold drop-in events in community settings, workplaces, and/or by mobile vans
Offer Pap testing at the same time as free testing for sexually transmitted infections
Offer clinic hours outside of regular workday
Reduce wait times at health departments and free clinics
Allow Medicaid patients to get a Pap test from someone other than their primary care provider
Access to a female doctor
Improve availability of interpreters
**Increase Provider Delivery (Provider Level)–Suggested by 7 women**
Remind the patient she is due for screening
Offer to provide the screening while the patient is already at the clinic
Instruct the patient at check-out to schedule an appointment for screening
Ask the overdue patient if she has barriers to screening, and attempt address them
**Increase Community Demand (Individual Level)–Suggested by 8 women**
Provide one-on-one education, including reasons for screening, what to expect, how to get it, and reassurance
Provide media-based education: provide information by videos, websites, or posters in doctors’ offices
Educate male partners about the importance of women’s health screenings
Widely advertise available resources for free and low-cost screening
Help Medicaid patients find providers accepting new Medicaid patients
Hold one-day events to increase motivation to "act now"
Offer "bring-a-friend" or other incentive

### Interventions to increase community access (economic/structural level)

The most common suggestion (n = 10) was to reduce out-of-pocket cost through expanded availability of free screenings, lower co-pays, and coupons. Payment plan options were also suggested. All ten women who suggested a cost-reduction approach were uninsured and eligible for free screening through the publicly funded Breast and Cervical Cancer Control Program (BCCCP), but were unaware of this resource. Five women suggested expanding insurance coverage and one suggested improving transportation options. Four women suggested ways to improve convenience, such as holding drop-in mobile clinics at community centers, workplaces, or low-income housing complexes, offering free Pap testing at the same time as free sexually transmitted infection screening, and reducing wait times at health departments and free clinics. Offering appointments outside regular work hours was suggested as helpful to women with jobs or in school. Five women with Medicaid expressed a desire to be able to go to a different provider for cervical cancer screening without having to change their primary care provider. Expanding Spanish-language services, including at appointment scheduling, was also suggested by the three Spanish-speaking participants.

### Interventions to increase provider delivery (provider level)

Two women wanted their providers to remind them of being due for screening. Three others, however, felt that a simple reminder was insufficient, and wanted their provider to tell them to get screened and provide clear instructions how to do so, such as by offering to perform the procedure at the same appointment or instructing the patient to schedule an appointment at checkout. They also suggested that the provider ask them why they had not been screened, so that barriers could be discussed and addressed if possible.

### Interventions to increase community demand (individual level)

Six women suggested a role for education to provide personalized information, reassurance, and motivation. Four women expressed a desire to have someone talk to them one-on-one to explain the screening procedure and reassure them, or to meet a woman affected by cervical cancer to help motivate them. One Latina participant recommended education for male partners on the importance of women’s health screenings. One suggested media-based education by videos, websites, or posters. The need to better advertise available resources such as BCCCP was noted, as was the need for assistance finding local providers accepting new Medicaid patients. Five women suggested increasing motivation to “act now” rather that put it off through one-day events in community locations, “bring a friend” offers, or time-limited incentives.

### Reactions to proposed interventions

**Fig 1:**
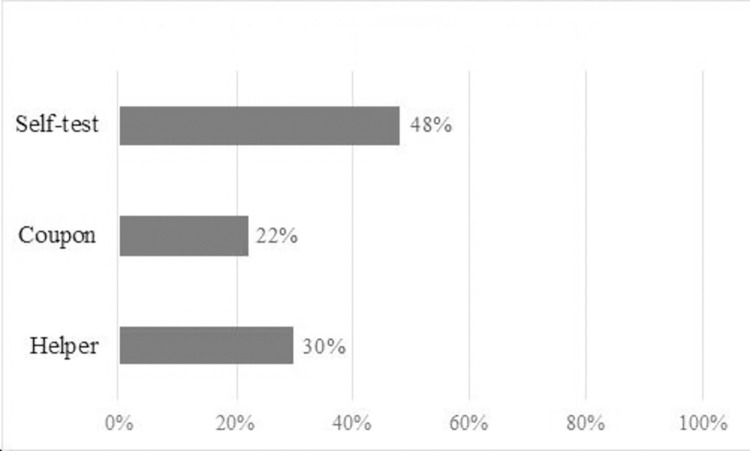
Intervention selected as “most helpful”.

Participants most frequently selected self-collection as most helpful (n = 11, 48%), followed by the helper (n = 7, 30%) and the coupon (n = 5, 22%) (Fig 1).

Two women suggested that a combination approach would be best: a website or phone number with information on screening guidelines, referral to clinics offering low-cost screening, and the opportunity to order an HPV self-test. Here we present feedback from all participants, not only those who preferred the given intervention (Table 5).

**Table 5:** Participant reactions to evidence-based interventions.

Barriers of women who preferred this intervention	Positive Reactions	Negative Reactions	Other Considerations
**One-on-one education (“Helper”)**			
Primary barriers: • Cost/ Trouble finding affordable provider • Does not seek preventative care in general• Low priority• Previous painful exam• Lack of provider recommendation or offer• Poor transportation• Embarrassment receiving from current providerAdditional barriers:• Low perceived need/ Lack of understanding of purpose• Discomfort receiving from unknown provider• Inconvenient hours and/or long waits at clinic• Fear of results	Personal attention increases perceived importance and motivation Personal education is a better way to learn: • More likely to remember a conversation than a flyer • Can ask questions and get feedback • Emotional support and personal experience • Particularly helpful to women who never screened Personalized assistance: • Address barriers “until there’s no reason not to do it” • Appointment scheduling for non-English speakers	Confidentiality concerns if not a medical professional (particularly if from within community) Feeling singled out or judged: “I would wonder why they were contacting me” Would not want to be approached in public place Education would not be helpful without removal of other barriers	Wide variation in comfort with the idea: • Some would be happy to talk to anyone with “good information” • Some were “very private” or “independent,” strongly averse to discussing the topic • Some would prefer if the “helper” were someone they knew, while others a preferred a stranger • Some liked the anonymity of a phone conversation, others felt they would be uncomfortable unless they had previously met the person Most liked the idea of an offer of same-day screening, but a some would want to have more advance warning for reasons of scheduling, “cleanliness,” or mental preparation
**Reducing Out-of-Pocket Costs (“Coupon”)**			
Primary barriers: • Cost • Trouble finding provider Additional barriers: • Low priority • Low perceived need/ Lack of understanding of purpose • Inconvenient hours and/or long waits at clinic Previous painful exam	Coupons are familiar and the sense of getting a discount can be motivating Low-risk to call a toll-free number and see what they have to offer: anonymous and free Wide reach If it was a good deal, women would tell each other about it	Many probably would not notice it, especially nor in the coupon section of the paper Easy to put off calling, put it in the “gonna do” pile No assistance with non-financial barriers Do not know anything about doctor’s experience, quality of care	Women who generally expressed more independence indicated that all they needed was information on where to go, therefore they tended to find this option sufficient
**Self-collection for HPV testing (“Self-test”)**			
Primary barriers: • Cost • Trouble finding affordable provider • Trouble finding Medicaid provider • Embarrassment due to male provider • Low priority • Discomfort receiving from unknown provider • Low perceived need Additional barriers: • Inconvenient hours and/or long waits at clinic • General low trust in screening • Lack of provider offer • Previous painful exam • Fear of result • Does not seek preventative care in general • Transportation • Weight	Avoid need for doctor’s appointment: • Save time and money • More likely to do it if quick and simple • Avoid potential pain • Avoid embarrassment: no one “looking at you” • No need to arrange transportation Would be interested in trying it for the novelty	Barriers remain if follow-up Pap smear is needed Concerns about: • Accuracy and confirming that sample was collected correctly • Hurting oneself doing “something a doctor’s supposed to do” • Missing out on a general pelvic exam, which could catch other things • Samples getting lost or damaged in the mail	Many had negative reactions until they understood that self-collection, detected a risk factor for cervical cancer, and was not a Pap On purchasing and kit dissemination: Some would pay $5–20. Some would order it for home delivery, but not purchase it in a store Participants stressed the importance of a hotline to answer questions and provide results

### Self-test (HPV self-collection)

The 11 women who selected the “self-test” as the most helpful cited convenience and privacy as the primary perceived benefits. Seven of these women named cost as their primary barrier to screening, and the others named trouble finding a provider, discomfort with a male or unknown provider, low priority, and low perceived need. Common perceived benefits were that self-collection could save time and money by avoiding a clinic visit and could be done with less pain and emotional discomfort. Some participants said that the novelty alone would be motivating. Negative reactions were concerns about accuracy, missing screenings for other health issues that could be detected by an in-person exam, and barriers to following up on a positive HPV result. Concerns about the accuracy of conducting self-collection for “something a doctor should do,” were often reduced when the interviewer explained that the self-collection device is designed to detect HPV infection, a risk factor for cancer, rather cancer itself. Five women raised the concern that if they received a positive HPV self-collection result, they would face many of the same barriers to follow-up (e.g., cost, transportation) that they faced to primary screening. Three women, however, believed a positive HPV result could increase their motivation to overcome their other barriers to completing in-clinic screening.

### Helper

The seven participants who preferred the helper intervention reported a wide range of primary barriers, from cost to general lack of preventive care to preference for a female doctor. Our description of the “helper” was intentionally non-specific, and the participants seemed to envision a role for the helper that best addressed their barriers, e.g., providing reassurance, helping with transportation, or making a referral to an affordable clinic. Some focused on personalized education, others described navigation through barriers, and many discussed both. Participants mentioned that they would be more likely to retain information provided in a face-to-face conversation than information in written materials, and that receiving personal attention would increase their motivation. However, some participants expressed that they would feel singled out or judged (n = 3), uncomfortable talking to a stranger (n = 4), or concerned about confidentiality (n = 2) if the helper were not a medical provider–particularly if this person was a member of their community. Four noted that if they were provided education without navigation, they would still face barriers of cost and transportation. We asked some women how they would react to being approached in a waiting room appointment and offered same-day screening: five women liked the idea, while seven expressed that they would want time to prepare mentally or to ensure personal “cleanliness”.

### Coupon

The five women who preferred the coupon identified cost as their primary barrier. None of the women who selected this option had expressed aversion to the testing procedure itself as a barrier, but rather lacked information on where to go or had not realized the importance of screening. Positive aspects included the familiarity of coupons, the motivating effect of receiving a discount, and the simplicity of distribution. Calling a hotline to get more information would be free, low-risk, and anonymous. Negative aspects identified were that cost reduction alone would not address other barriers, coupons might not be noticed, and referral to an unknown provider could introduce concerns about quality of care. Overall, this option appealed most to women whose only barriers were perceived cost and difficulty finding an affordable provider.

## Discussion

Our findings support evidence that simultaneously addressing multiple barriers may be needed to substantially increase cervical cancer screening among infrequently screened women. Cost was a common primary barrier to completing screening in this group of women. Nearly all participants reported multiple barriers to screening. Participants most commonly selected HPV self-collection as “most helpful” to them to undergo screening compared to one-on-one education or reducing out-of-pocket costs, but noted that barriers to follow-up of an abnormal screening result would remain.

Other research has similarly found that cost is a major barrier to screening for uninsured women [29]. Evidence suggests that interventions reducing out-of-pocket costs, such as through vouchers, reducing copays, or adjusting insurance coverage, can increase uptake of breast and cervical cancer screening [30, 31]. Most participants in our study who reported cost as their primary barrier were eligible for free screening through BCCCP and at free clinics in the area. Raising awareness about and navigation to such free services may be needed to maximize the impact of providing low cost screenings. It is worth noting that several women reported that they would probably be willing to find the money to screen if it were not for other barriers (e.g., if Pap tests were not uncomfortable). Indeed, our finding that nearly all women reported multiple barriers to screening suggests that multiple or multicomponent interventions may be necessary to make a substantial impact on cervical cancer screening rates.

Evidence suggests that multicomponent interventions–those that combine two or more evidence-based interventions–have a greater effect on increasing cancer screening than single component interventions, and are cost-effective to increase cervical cancer screening [28, 32]. For example, a multicomponent intervention with Korean American women that combined community education with individual navigation and a reminder letter attained 72% Pap test screening rates compared to 10% in the education-only control arm (*p<*0.001) [33]. A CPSTF review found the largest effects from interventions that combined approaches at all levels (community demand, provider delivery, and community access–median increase of 24 percentage points), and the second largest among those that combined interventions at the community access and demand levels (median 11 point increase) [28].

Interventions suggested by participants were largely consistent with those recommended by current evidence. For example, a review of “contextual factors” (encompassing provider and systems characteristics) associated with breast and cervical cancer screening uptake found that provider recommendation, female providers, flexible appointment times, patient reminders, and organizational evaluation of provider performance were all associated with higher screening rates [11]–each of which was suggested by one or more participants in our study.

HPV self-collection is a promising potential intervention that received positive feedback from participants. Advantages reported by our participants are consistent with the findings of self-collection studies, which find commonly reported reasons for preferring HPV self-collection to pelvic exam-based screening including ease of use, reduced embarrassment, privacy, comfort, and convenience [21, 34]. Concerns about performing self-collection incorrectly have also been reported previously [14], including in approximately one fifth of participants in a recent meta-analysis of self-collection trials [34]. Women reported being less concerned about self-collection after our study interviewer provided additional information about HPV testing, which highlights the role for education to support a self-collection intervention. Given the finding that most women experienced multiple barriers to screening, factors such as cost, patient education, and navigation to follow-up care should be considered to maximize the impact of a self-collection intervention [14].

One strength of our study is that our participants comprised the target population for cervical cancer screening interventions by virtue of being overdue for screening, and included individuals from groups at elevated risk for developing cervical cancer: black race, low-income, living in a county with low economic development, with family history of cervical cancer, uninsured, and publicly insured. Second, by describing the interventions in general terms, we were able to elicit participants’ own interpretations of the interventions. In practice, there is considerable diversity in the interventions that are evaluated in systematic reviews and categorized into a particular type of evidence-based intervention [35, 36]. For example, the CPSTF definition of “small media” encompasses everything from videos to posters to newsletters delivered in many different ways with different content and messages [37].

Among study limitations, convenience sampling and self-selection into the study may have resulted in a sample that was more interested in cervical cancer screening than the general population of women overdue for screening. There is a potential in qualitative research for reactive self-report, by which a participant provides a response in line with what they think the interviewer wants to hear [26]. We attempted to minimize this effect by telling the participants, “I didn’t come up with these ideas, so you don’t need to worry about hurting my feelings,” and participants did provide substantial negative feedback on proposed interventions. Counts must be interpreted with some caution, given the small sample size and non-representative sampling. However, by collecting detailed feedback on each intervention and the complexities of barriers, the study provides insights into participant preferences and experiences beyond simple rankings. Finally, passage of time since collection of the data may have resulted in some changes to relevant contexts. For example, the relevance of cost as a factor may be less relevant following implementation of the Patient Protection and Affordable Care Act (ACA) over the past decade. However, in 2020, 27.5 million adults aged 18–64 (13.9%) remained uninsured nationwide [38], and low-income individuals in states, like North Carolina, that have not expanded Medicaid, and Black, Indigenous, and people of color (BIPOC) individuals are more likely to be uninsured [39].

## Conclusions

Study findings suggest that women experience multiple and interacting barriers to screening. Accordingly, multicomponent interventions may be required to make significant difference in cervical cancer screening uptake. Self-collection for HPV testing is a promising intervention to increase screening uptake. Attention must be paid to assist women to overcome barriers to follow-up on an abnormal screening result, and on messaging about the self-collection process and meaning of HPV self-collection results to maximize its benefits.
